# PsycoLaus: interest of a joint physical and psychiatric investigation in the community

**DOI:** 10.1186/1753-6561-7-S5-O18

**Published:** 2013-08-30

**Authors:** M Preisig

**Affiliations:** 1 Department of Psychiatry, University Hospital Centre and University of Lausanne, Switzerland

## 

Given the high prevalence and serious consequences of both CVD and mental disorders, associations between the two types of diseases would have a major impact on public health. If there is a causal link between CVD and mental disorders, the screening and early treatment of one of the two types of diseases is likely to have a preventive effect on the other pathology. The mechanisms underlying this co-morbidity is poorly understood, partially due to major limitations of existing studies such as the use of clinical samples, the lack of an appropriate comparison group, the use of psychiatric rating scales for a single psychiatric syndrome rather than structured diagnostic interviews, reliability on self-reported somatic information rather than objective measurements and a lack of simultaneous assessment of cardiovascular risk factors which does not allow the study of potential pathways from a specific mental disorder to cardiovascular disease.

The CoLaus/PsychoLaus is a prospective follow up of participants in the CoLaus cohort to assess whether mental diseases increase vulnerability to cardiovascular disease/risk factors or vice versa and to identify common pathogenic processes involved. It also gives better knowledge about long term course of mental disorders and cardiovascular diseases and service utilisation patterns all of which can result in more effective strategies in prevention and treatment. Current evidence from CoLaus/PsyCoLaus suggests a significant role of a typical depression symptom, which are strongly associated to physical and behavioural cardiovascular risk factors.

